# Educational technologies on signs of gestational risk and/or labor for pregnant women: a scoping review*

**DOI:** 10.1590/1980-220X-REEUSP-2025-0188en

**Published:** 2025-08-29

**Authors:** Thaís Jormanna Pereira Silva, Ana Carolina Nunes de Macêdo, Caroline Magalhães de Alcântara, Maria Veraci Oliveira Queiroz, Igor Cordeiro Mendes, Thaís Marques Lima, Juliana Alencar Moreira Borges

**Affiliations:** 1 Universidade Estadual do Ceará, Programa de Pós-Graduação em Cuidados Clínicos em Enfermagem e Saúde, Fortaleza, CE, Brazil.; 2 Universidade Federal do Rio Grande do Norte, Faculdade de Ciências da Saúde do Trairi, Santa Cruz, RN, Brazil.

**Keywords:** Educational Technology, Prenatal Care, Health Education, Pregnancy Complications, Labor, Obstetric

## Abstract

**Objective:**

To map educational technologies on signs of gestational risk and/or labor developed or used to guide pregnant women in the context of Brazilian prenatal care.

**Method:**

Scoping review conducted in accordance with JBI with searches performed in the following databases: Scopus, Web of Science, PubMed/MEDLINE, LILACS, BDENF, BDTD, and Google Scholar, without time restriction. Data selection and extraction in independent stages by pairs, and narrative synthesis according to the PAGER model.

**Results:**

Twenty-one studies were included, which described 18 technologies: 10 digital (55.5%), 8 printed (44%), and 1 audiovisual (5.5%). The majority (n = 13; 72.2%) approached the signs in a complementary way. There was a predominance of resources with accessible language, instructive elements, and the involvement of Nursing in their design. Thematic, methodological, and geographical gaps were observed, especially in the exclusive focus on signs of gestational risk, diversity of participants, and use of realistic images.

**Conclusion:**

Mapped technologies highlight important advances, as well as the need to develop more specific, inclusive, and clinically validated tools. Such resources can qualify prenatal practices in Nursing and health and improve maternal and neonatal outcomes.

## INTRODUCTION

Educational Technologies in Health (ETH) encompass strategies and resources aimed at promoting health knowledge for different audiences, aiming to facilitate understanding and promote self-care^(1)^. In obstetrics, ETH have been consolidating themselves as relevant support for strengthening maternal and child health, especially in the context of prenatal, childbirth, and postpartum care^(2)^.

Regarding the topics covered in the maternal and child area, review studies highlight the predominance of guidelines on healthy eating, preparation for childbirth, obstetric violence, breastfeeding, postpartum care, and newborn care^(2, 3, 4)^, with limited attention to guidance on signs of risk during pregnancy and the onset of labor^(5)^. This gap is worrying given the persistence of challenges in Brazilian obstetric public health, such as high maternal mortality^(6)^.

To seek timely assistance, it is essential that pregnant women recognize the signs of imminent or ongoing labor (regular contractions, loss of the mucous plug, rupture of the waters) and the signs that indicate gestational risk (vaginal bleeding, intense headache, blurred vision, persistent abdominal pain, absence of fetal movements, changes in vaginal fluid, fever). However, studies indicate that only 39% of Brazilian pregnant women are able to correctly identify most of these signs, revealing an urgent need for specific educational strategies in prenatal care^(5)^.

The development and use of ETH in the obstetric context are in line with national guidelines for strengthening the Maternal and Child Care Network, such as the *Rede Cegonha* and the *Rede Alyne*, by expanding access to qualified information and favoring safe decision-making by pregnant women. At a global level, these technologies also contribute to achieving the Sustainable Development Goal (SDG) of the United Nations 2030 Agenda, which aims to ensure healthy lives and promote well-being for all, at all ages^(7)^.

Before formulating this review, a preliminary search was carried out to verify the existence of review studies and registered protocols that answered the question of the present investigation. This search was conducted on the PROSPERO, Open Science Framework (OSF), PubMed, and Scopus platforms. Reviews with different scopes were found^(2, 3, 4)^, not specifically considering ETH on signs of gestational risk and labor in the Brazilian scenario, which reinforces the originality and relevance of the study.

Therefore, this study aimed to map educational technologies on signs of gestational risk and/or labor developed or used to guide pregnant women in the context of Brazilian prenatal care.

## METHOD

### Design of Study

This is a scoping review carried out in nine stages according to the JBI Manual (2024) and following the guidelines of the Preferred Reporting Items for Systematic Reviews and Meta-Analyses extension for Scoping Reviews (PRISMA-ScR)^(8)^. The protocol was registered on the OSF platform (DOI: 10.17605/OSF.IO/24JW7).

### Population and Selection Criteria

The research question was structured based on the PCC strategy, in which the population is pregnant women; the concept, ETH aimed at providing guidance on signs of risk during pregnancy and signs of labor; and the context, Brazilian prenatal care, regardless of the level of care. In view of this, the question was defined: What educational technologies have been developed or used in the context of Brazilian prenatal care to guide pregnant women about signs of gestational and labor risk?

### Sample Definition

Studies in Portuguese, English or Spanish, with full text, without time restrictions, addressing ETH on signs of gestational and/or labor risk in the context of Brazilian prenatal care were included. Studies that did not include these signs, were aimed exclusively at other audiences, or were reviews of any nature were excluded.

### Data Collection

The search strategy was developed based on the recommendations of the *JBI* (2024) and constructed by the authors, who have previous experience in scoping reviews. Controlled descriptors (DeCS and MeSH) and free terms were used, duly adapted to the logic of each database. Searches were carried out in the Scopus, Web of Science, PubMed/MEDLINE, LILACS, BDENF, BDTD and Google Scholar databases, with access to the first three through the CAPES/CAFe Portal. In international databases, the term “Brazil” was added to delimit the national context of the investigation. The strategies are detailed in Chart 1.

**Chart 1:** Search strategies used on databases – Fortaleza, Ceará, Brazil, 2025.

**LILACS and BDENF**
(“Educação em Saúde” OR “Tecnologia Educacional” OR “Material Educativo” OR “Recursos Educacionais”) AND (“Gravidez” OR “Gestantes” OR “Cuidado Pré-Natal”) AND (“Complicações na Gravidez” OR “Trabalho de Parto” OR “Parto”)
**Digital Library of Theses and Dissertations (BDTD)**
(“tecnologia educativa” OR “tecnologia educacional” OR “material educativo”) AND (“pré-natal” OR “gestação” OR “sinais de risco” OR “trabalho de parto” OR “parto”)
**Google Scholar**
(“tecnologia educativa” OR “tecnologia educacional” OR “material educativo” OR “recurso educativo” OR “estratégia educativa”) AND (“pré-natal” OR “gravidez” OR “gestantes”) AND (“sinais de risco” OR “complicações na gestação” OR “alertas na gravidez” OR “trabalho de parto” OR “sinais de parto”)
**PubMed/MEDLINE**
(“Health Education”[MeSH] OR “Educational Technology”[MeSH]) AND (“Pregnancy”[MeSH] OR “Prenatal Care”[MeSH] OR “Labor, Obstetric”[MeSH] OR “Pregnancy Complications”[MeSH]) AND (Brazil[MeSH])
**Scopus**
TITLE-ABS-KEY (“health education” OR “prenatal education” OR “educational technology”) AND (“pregnancy” OR “prenatal care” OR “labor” OR “childbirth” OR “pregnancy complications”) AND (“Brazil”)
**Web of Science**
TS = ((“health education” OR “prenatal education” OR “educational technology”) AND (“pregnancy” OR “prenatal care” OR “labor” OR “pregnancy complications”) AND (“Brazil”))

### Data Analysis and Treatment

Study screening was performed on the Rayyan platform, in two stages: titles/abstracts reading and complete reading, following defined criteria. Two reviewers acted independently. In case of disagreement, a third reviewer was expected to act, although this was not necessary. After screening, the included studies were subjected to full reading.

Data were extracted using a form prepared by the authors containing information such as title, year, federative unit of publication, type of publication, type and characteristics of technology, content covered and form of presentation, types of images, target audience, validation stage, and participants involved, among others. It should be noted that the images were classified by type: illustrations and photographs, and by purpose: decorative, with no direct relation to the content, and instructive, used to explain or exemplify the signs covered^(9)^. The form was previously tested in three studies for verification of consistency and adequacy of the fields.

The description of the results was conducted according to the methodological guidelines for scoping reviews, respecting the exploratory and non-evaluative nature of this type of study^(8)^. To support the discussion of the results, the PAGER model (Patterns, Advances, Gaps, Evidence for Practice, and Research Recommendations) was adopted, which guided the analysis in five dimensions: identified patterns, advances, gaps, evidence for practice, and recommendations for future research^(10)^.

### Ethical Aspects

This scoping review used exclusively publicly available secondary data and, therefore, did not require approval by a Research Ethics Committee. All stages were conducted in accordance with the ethical principles of methodological rigor, fidelity in the reproduction of information and adequate attribution of sources.

## RESULTS

**Figure 1:**
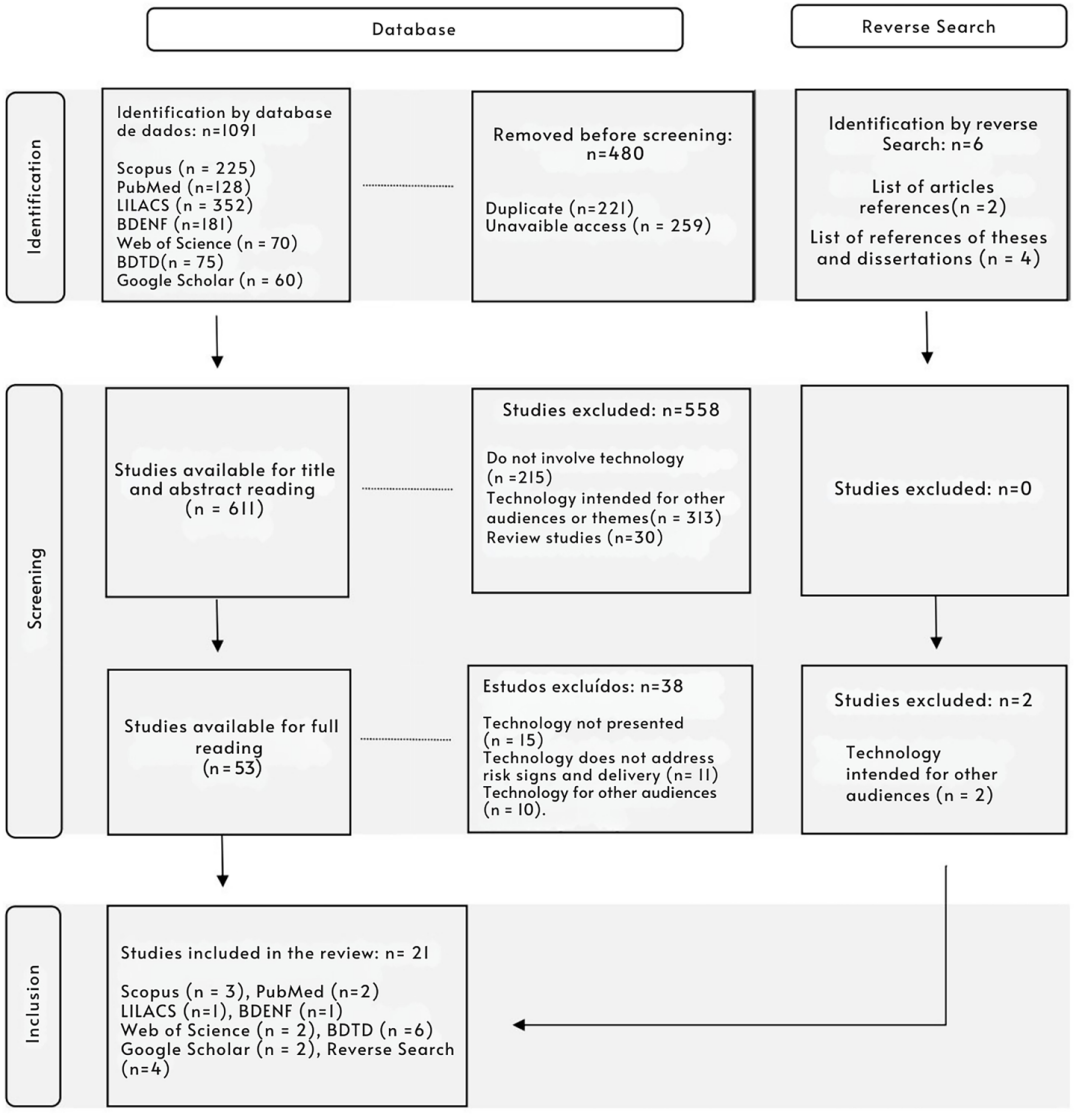
Flowchart of screening and inclusion of studies according to PRISMA-ScR.

Source: Adapted from Preferred Reporting Items for Systematic Reviews and Meta-Analyses Extension for Scoping Reviews, 2020.

The search in seven databases resulted in the identification of 1089 records, of which, after screening and eligibility analysis, 21 studies were included. The PRISMA-ScR flow diagram, presented in Figure 1, illustrates this selection process in detail.

The majority of studies (62%) corresponded to articles published in national Nursing journals; the others were dissertations (28.5%) and theses (9.5%). The highest concentration of publications occurred between 2019 and 2022, with emphasis on the year 2022. Regarding the design, 12 studies were methodological (57%), seven clinical trials (33%), four controlled and randomized (19%) and three quasi-experimental single-group studies (14%), in addition to two experience reports (10%). Additional information is available in Chart 2.

**Chart 2:** Characterization of included studies according to year, status, publication format, journal, methodological type, and professional authorship – Fortaleza, Ceará, Brazil, 2025.

Study title	Year	State	Format Journal	Design of study	Authors’ occupation
Cartilha educativa sobre trabalho de parto e parto: estudo de validade^(11)^	2024	MS	Article (Rev. Bras. Enferm.)	Methodological	Nurse
Conhecimento, atitude e prática sobre síndrome hipertensiva gestacional entre gestantes: ensaio clínico randomizado^(12)^	2022	CE	Article (Texto Contexto Enferm)	Clinical trial	Nurse
Validação de tecnologia educativa para mulheres indígenas sobre o pré-natal^(13)^	2022	CE	Thesis	Methodological	Nurse
Desenvolvimento do álbum seriado meu bebê vai nascer: guia da gestante para o trabalho de parto, parto e pós-parto^(14)^	2019	CE	Dissertation	Clinical trial	Nurse
Contribuição de gestantes na construção e avaliação de uma tecnologia educacional: o “Almanaque da Gestante”^(15)^	2021	ES	Article (Rev. Bras. Enferm.)	Methodological	Nurse Graphic designer
Simplesmente mães: construção compartilhada de tecnologias sobre pré-natal de mulheres com deficiência visual^(16)^	2024	PA	Article (Cogitare Enferm)	Methodological	Nurse
Quando ir para a maternidade? Educação em saúde sobre o trabalho de parto^(17)^	2017	MG	Article (Rev enfermUFPE)	Experience Report	Nurse Physician
Infográfico para gestantes e acompanhantes: tecnologia educacional no contexto do atendimento obstétrico^(18)^	2023	SC	Article (Cogitare Enferm)	Methodological	Nurse
Desenvolvimento de aplicativo móvel para o acompanhamento pré-natal e validação de conteúdo^(19)^	2022	RN	Article (Acta Paul Enferm)	Methodological	Nurse
Eficácia de aplicativo móvel na adesão de gestantes às consultas de pré-natal: ensaio clínico randomizado^(20)^	2021	RN	Article (Rev. Bras. Enferm.)	Clinical trial	Nurse
Intervenção Educativa no Cuidado Obstétrico através de um Aplicativo para Dispositivos Móveis: APP^(21)^	2018	MG	Article (Rev. Intern. em Líng. Portuguesa)	Experience Report	Physician Computer engineer Systems analyst
Intervenção educativa para conhecimento, atitude e prática de gestantes adolescentes no preparo para o parto vaginal: ensaio clínico randomizado^(22)^	2022	PE	Dissertation	Clinical trial	Nurse
SABER G-ESTAR: construção e validação de um aplicativo móvel para educação em saúde no ciclo gravídico-puerperal^(23)^	2021	RJ	Thesis	Methodological	Nurse
Desenvolvimento e validação de um protótipo de aplicativo para plataforma móvel para promoção da saúde de gestantes^(24)^	2019	CE	Thesis	Methodological	Nurse
Desenvolvimento de um protótipo de software para monitoramento de gestantes^(25)^	2019	AL	Thesis	Methodological	Nurse
Implementação de uma tecnologia de informação aplicada ao acompanhamento e promoção do cuidado à gestante^(26)^	2020	BA	Thesis	Methodological	Medicina
Desenvolvimento de website para gestantes: estudo metodológico^(27)^	2022	AC	Thesis	Methodological	Nurse
Avaliação de objeto virtual de aprendizagem sobre pré-natal para adolescentes grávidas na atenção básica^(28)^	2020	PI	Article (Acta Paul Enferm)	Methodological	Nurse
Efeito de intervenção educativa online na qualidade de vida de gestantes adolescentes^(29)^	2022	PI	Article (Acta Paul Enferm)	Clinical trial	Nurse
Estratégia de comunicação e informação em saúde e percepção de sentir-se preparada para o parto: ensaio aleatorizado por conglomerados^(30)^	2019	SP	Article (Cad. Saúde Pública)	Clinical trial	Enfermagem Medicina Jornalismo Nutrição Psicologia Fisioterapia Biomedicina
Tecnologia educacional para primigestas: estudo quase experimental^(31)^	2022	RN	Article (Rev. esc. nurs. USP)	Clinical trial	Nurse

These studies described 18 technologies, developed or used in the context of Brazilian prenatal care, between 2017 and 2024. It should be noted that three of these technologies (T9, T10 and T16) were addressed in two distinct studies, with complementary approaches, such as construction and validation in one, and effectiveness evaluation in the other.

Geographically, the technologies were mostly developed in the Northeast (50%) and Southeast (27.8%), with less representation from the North, South and Central-West regions.

Chart 3 presents a summary of the mapped educational technologies, organized according to format (printed materials, digital resources, and audiovisual resources), and includes information on the signs covered and the form of presentation used in each resource.

**Chart 3:** Organization of educational technologies mapped according to types, signs of labor, signs of risk and form of presentation – Fortaleza, Ceará, Brazil, 2025.

Technology			Signs of		Presentation form
			Delivery	Risk	
**Printed Materials (44%)**	T1	Handbook *Cada parto é uma história* (Each birth is a story^(11)^	■	□	Text, decorative and instructive illustrations, photographs
	T2	Handbook HDP Prevention^(12)^	□	■	Instructive text and illustrations
	T3	Handbook on Care for Pregnant Indigenous Women^(13)^	■	■	Text and decorative illustrations
	T4	Serial album *Meu bebê vai nascer* (My baby is going to be born)^(14)^	■	■	Instructive text and illustrations
	T5	Pregnancy almanac^(15)^	■	■	Comics, word searches, crosswords
	T6	Guide *Simplesmente Mães* (Simply Moms)^(16)^	■	■	Text adapted for screen readers.
	T7	Folder When to go to the maternity ward?^(17)^	■	■	Text, decorative illustrations
	T8	Obstetric infographic^(18)^	■	■	Topical texts, symbols, and instructional illustrations
**Digital Resources (50%)**	T9	Healthy Pregnancy App^(19,20)^	■	■	Text in interactive menus
	T10	My Prenatal App^(21,22)^	■	■	Text in Q&A, animated videos
	T11	*Saber G-estar* App^(23)^	■	■	Text, instructive and decorative illustrations
	T12	App *Gestar* ^(24)^	■	■	Instructive and decorative text and illustrations
	T13	App *Mitera* ^(25)^	■	■	Interactive quiz, icons
	T14	Webapp Together in prenatal care^(26)^	■	■	Text in questions and answers
	T15	Website My Birth^(27)^	■	□	Instructive text and illustrations
	T16	GESTAQ Virtual Learning Object^(28,29)^	■	□	Text, video, interactive forums
	T17	PRENACEL Short Text Messaging System^(30)^	■	■	SMS Text Messages
**Audiovisual Resources (6%)**	T18	Video When to go to maternity?^(31)^	■	■	Text, audio, instructional illustrations and Libras

Legend: ■ Approached signal; □ Not approached signal.

In ten technologies (55.5%), signs of gestational and/or labor risk were addressed in a complementary manner, inserted into broader proposals for education in the pregnancy-puerperal cycle. Only five (27.8%) ETH focused exclusively on providing guidance on signs of gestational risk and/or signs of labor.

**Figure 2:**
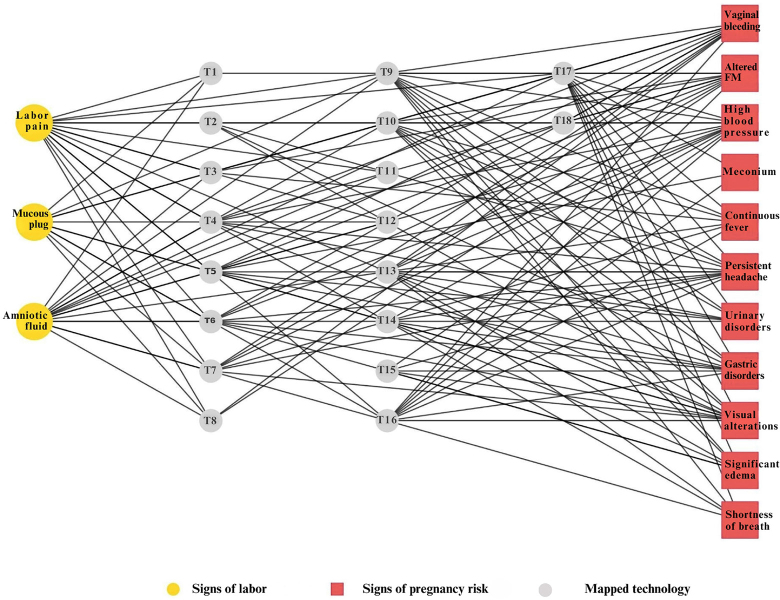
Network diagram of connections between educational technologies, signs of labor, and signs of gestational risk.

Figure 2 presents the network diagram that summarizes the connections between the educational technologies included and the content covered. The thematic distribution is observed among those that deal exclusively with signs of gestational risk, signs of labor, or both, allowing us to visualize the scope and focus of each mapped technology.

Regarding the validations to which the mapped technologies were submitted, these are described in Chart 4, covering quality validation, which includes aspects such as content, appearance, and usability, and clinical validation, aimed at analyzing the effects on the target audience. The results refer to quality validation, and the outcomes to clinical validation.

**Chart 4:** Summary of validation, results, and outcomes of educational technologies mapped in the scoping review – Fortaleza, Ceará, Brazil, 2025.

Technology	Validation type	Results/Outcomes
T1^(11)^	Content and appearance	High CVI = 0.91 and good internal consistency (Cronbach’s alpha > 0.8) in the experts’ assessment. Considered understandable and useful by 95.5% of pregnant women.
T2^(12)^	Content, appearance and clinical approach	Validation by experts. Further information not provided. Significant improvements in the KAP of pregnant women on HDP (p < 0.05).
T3^(13)^	Content and appearance	It achieved excellent reliability and satisfaction among experts (CVI = 0.95).
T4^(14)^	Content, appearance and clinical approach	Considered easy to understand by pregnant women (AI = 99%) and high acceptance by experts (CVI = 0.92, Cronbach’s alpha = 0.9). Positive impact on pregnant women’s knowledge about labor, delivery and postpartum (p < 0.05).
T5^(15)^	Content and appearance	Considered by pregnant women to be easy to understand, self-explanatory, aesthetically attractive and motivating for good practices (AI = 92.1%).
T6^(16)^	Qualitative by interview	Positively evaluated by the target audience, for meeting specific demands and promoting autonomy.
T7^(17)^	Content	Adapted by experts and distributed during consultations to provide information on warning signs and childbirth.
T8^(18)^	Content and appearance	Considered suitable for dissemination of information by experts; CVI of 83.3% after two Delphi rounds.
T9^(19,20)^	Content, usability and clinical approach	Considered valid by nurses and IT professionals with consensus level of 83.3% after two Delphi rounds. High rate of adherence to prenatal care among pregnant women in the intervention group (83.3% vs. 7.7% in the control group; p < 0.05).
T10^(21,22)^	Content, usability and clinical approach	Positively evaluated by experts and pregnant women. It favored KAP of adolescents on preparation for childbirth.
T11^(23)^	Usability and acceptance	Significant level of satisfaction among pregnant women (CVI = 0.98 and Kappa = 0.96)
T12^(24)^	Content, usability and appearance	Prototype well accepted among experts, CVI: content (0.68), usability (0.62), appearance (0.93).
T13^(25)^	Usability and appearance	Easy handling for 87.5% of pregnant women; favored the perception of risk symptoms.
T14^(26)^	Qualitative by interview	Well accepted by users, especially first-time mothers
T15^(27)^	Content, appearance and usability	Agreement greater than 0.8 between experts and pregnant women in the evaluations of content, appearance and usability.
T16^(28,29)^	Content, usability, functionality	Ratings ≥ 80% in the dimensions of quality, functionality and efficiency by experts. Positive impact on the quality of life of pregnant adolescents (p < 0.001).
T17^(30)^	Content and clinic	Validation by pregnant women and specialists. Further information not provided. Use associated with greater perception of preparation for childbirth.
T18^(31)^	Content and clinic	High satisfaction among first-time mothers (average of 9.92) Significant increase in knowledge about clinical signs after intervention

Legend: CVI = Content Validation Index; AI = Agreement Index; CAP = Knowledge Practical Attitude.

## DISCUSSION

The analysis of the 18 mapped ETH revealed a predominance of digital resources, reflecting contemporary trends in the field of health education, with emphasis on greater accessibility, information reach, and the possibility of agile content updating^(23,24,31)^.

### Patterns – Identified Patterns

A pattern of clear, accessible, simple and culturally appropriate language was observed, combined with the use of instructive illustrations to explain or exemplify the signs of risk and aspects of labor. Some ETH used playful strategies such as games and comics^(15)^, storytelling^(14,28,31)^, forums and communication channels with health professionals, promoting engagement and strengthening the bond between pregnant women and care teams^(19,28,30)^.

### Advances – Observed Advances

Advances were evidenced in the methodological rigor adopted in the construction of the ETH, with a strong basis in updated theoretical references and validation by specialists and/or pregnant women. Technologies developed based on the needs of the target audience stand out^(13,15,16,18,19,23)^, a crucial factor in increasing acceptability and effectiveness.

Furthermore, clinical validations have reinforced the credibility of some technologies, indicating positive results such as increased knowledge about signs of risk and labor^(14,31)^, greater perception of preparation for childbirth^(30)^, and significant improvements in KAP about HDP^(12)^ and the delivery^(22)^, enhancing credibility and favoring the replicability of these technologies.

### Gaps – Identified Gaps

Despite the advances, relevant gaps were identified. Few technologies have exclusively addressed signs of gestational risk or labor, and it is common for these contents to be included in a secondary way in broader educational proposals, which can compromise their effectiveness in situations requiring rapid decision-making^(12,17,18,25,31)^.

A shortage in the use of realistic visual resources, such as photographs, which are potentially more effective in representing clinical signs, was also observed^(11)^. Furthermore, scientific production showed geographic inequality, being concentrated mainly in the Northeast^(12, 13, 14,19, 20, 21,24, 25, 26,28,29,31)^ and Southeast^(15,17,21,23,30)^ regions, with little representation from regions such as the North, marked by greater access challenges, cultural diversity, and worse obstetric indicators^(27)^.

Another critical aspect was the limitation in the representation of participants in the construction and validation processes. Participation was concentrated in the regions where the studies were conducted, which restricts cultural and linguistic diversity. Some authors reported difficulty in bringing together judges from different states^(11)^ and low participation of invited evaluators^(19,27)^. Furthermore, the inclusion of pregnant adolescents (10 to 19 years old) was little explored, despite their greater vulnerability to misinformation and insecurity regarding pregnancy and childbirth^(13,14,22)^.

### Evidence for Practice – Implications for Practice

The findings of this review provide relevant support for improving public policies aimed at maternal and child health, especially within the scope of the Brazilian Public Health System (*SUS*). The mapped EHT presented promising evidence to qualify prenatal care, promote early recognition of critical signs, and strengthen the autonomy of pregnant women, so that their use as a complement to professional practice can contribute to the reduction of adverse maternal and neonatal outcomes.

Therefore, the incorporation of these technologies into prenatal educational actions, care protocols and strategies of the *Rede Cegonha* and *Rede Alyne* represents a potential opportunity to promote more responsive, inclusive, and evidence-based care.

The active involvement of Nursing in the development and application of these technologies reinforces the strategic role of the profession in promoting maternal and child health and educational innovation^(11, 12, 13, 14, 15, 16, 17, 18, 19, 20,22, 23, 24, 25,27, 28, 29, 30, 31)^. Initiatives that incorporate accessibility, such as the guide aimed at women with visual impairments^(16)^ and the video with translation into the Brazilian Sign Language (Libras)^(31)^, deserve to be highlighted as practices that promote equity in access to information.

### Recommendations for Future Research

It is recommended that new technologies be developed that expand the use of realistic visual resources, such as photographs, associated with explanatory illustrations, respecting the sociocultural context and sensitivity of the topic. This combination can enhance the understanding of clinical signs, especially in audiences with different levels of health literacy^(9, 10, 11,22)^.

To overcome the identified geographical limitations, it is important to decentralize the production and validation processes of technologies, and involve participants from different regions of the country. The inclusion of diverse realities can expand the applicability and cultural sensitivity of educational tools, contributing to their effectiveness in multiple contexts. To this end, it is recommended that partnerships be formed between research institutions from different regions, on a collaborative or multicenter basis, favoring sample diversity and methodological sharing. Complementary strategies for attracting participants may include the use of social media, dissemination through scientific and professional associations, expansion of contact channels (email, WhatsApp, telephone calls), sending of automated reminders, and validation of the viability of established deadlines for feedback, to favor engagement, co-responsibility, and retention of participants throughout the process.

In the case of adolescents, active inclusion as participants in the processes of construction and evaluation of technologies should be encouraged^(22,28,29)^. To this end, it is suggested that there be coordination with schools, primary health units and specialized services in sexual and reproductive health, which can facilitate recruitment, engagement, and qualified listening of this group.

Finally, the importance of carrying out rigorous clinical validations is reinforced, with robust designs and assessment of clinical and behavioral outcomes, such as knowledge, attitude, practice, and adherence to prenatal care. These results are essential to support the adoption of technologies in prenatal care and their incorporation into evidence-based public policies^(12,14,20,22,29)^.

### Study Limitations

Although the BDTD was used as a source of grey literature, the non-systematic inclusion of other non-indexed institutional repositories may have limited the identification of educational technologies. Furthermore, although the search strategies were broad and conducted rigorously, it is possible that the selection of databases and descriptors limited the identification of relevant studies.

## CONCLUSION

This scoping review mapped 18 educational technologies developed or used in the context of Brazilian prenatal care to guide pregnant women on signs of gestational risk and signs indicative of labor.

The results show progress in the incorporation of digital resources, accessible language, and rigorous validations, with the potential to improve prenatal care and strengthen the autonomy of pregnant women. However, thematic, methodological and geographical gaps persist, including the limited exclusive approach to critical signs, low regional representation, and the scarce inclusion of pregnant adolescents.

The findings contribute to nursing science by indicating paths for the development of more specific, inclusive and clinically validated technologies, capable of supporting evidence-based care practices and positively impacting maternal and neonatal outcomes.

The need for future research focused on expanding the cultural diversity of the audiences involved and the use of more realistic visual resources to enhance the effectiveness of educational interventions is reinforced.

## Data Availability

All datasets supporting the findings of this study are available upon request from the corresponding author.
