# Supplementary material for: Educational technologies on signs of gestational risk and/or labor for pregnant women: a scoping review
Source: Rev Esc Enferm USP. 2025 Aug 29;59:e20250188. doi: 10.1590/1980-220X-REEUSP-2025-0188en (PMC12395603; doi:10.1590/1980-220X-REEUSP-2025-0188en)
Supplement: Translation

# Translation: Tecnologias educativas sobre sinais de risco gestacional e/ou trabalho de parto para gestantes: revisão de escopo*

**DOI:** 10.1590/1980-220X-REEUSP-2025-0188pt

**Authors:** Thaís Jormanna Pereira Silva, Ana Carolina Nunes de Macêdo, Caroline Magalhães de Alcântara, Maria Veraci Oliveira Queiroz, Igor Cordeiro Mendes, Thaís Marques Lima, Juliana Alencar Moreira Borges

**Affiliations:** 1 Universidade Estadual do Ceará, Programa de Pós-Graduação em Cuidados Clínicos em Enfermagem e Saúde, Fortaleza, CE, Brasil.; 2 Universidade Federal do Rio Grande do Norte, Faculdade de Ciências da Saúde do Trairi, Santa Cruz, RN, Brasil.

**Keywords:** Tecnologia Educacional, Cuidado Pré-Natal, Educação em Saúde, Complicações na Gravidez, Trabalho de Parto

## Abstract

**Objetivo:**

Mapear tecnologias educativas sobre sinais de risco gestacional e/ou trabalho de parto desenvolvidas ou utilizadas para orientar gestantes no contexto do pré-natal brasileiro.

**Método:**

Revisão de escopo conduzida conforme o JBI com buscas realizadas nas bases: Scopus, Web of Science, PubMed/MEDLINE, LILACS, BDENF, BDTD e Google Scholar, sem restrição temporal. Seleção e extração dos dados em etapas independentes por pares, e síntese narrativa conforme o modelo PAGER.

**Resultados:**

Incluídos 21 estudos, que descreveram 18 tecnologias: 10 digitais (55,5%), 8 impressas (44%) e 1 audiovisual (5,5%). A maioria (n = 13; 72,2%) abordou os sinais de forma complementar. Observou-se predomínio de recursos com linguagem acessível, elementos instrutivos e envolvimento da Enfermagem em sua concepção. Lacunas temáticas, metodológicas e geográficas foram identificadas, especialmente no foco exclusivo sobre sinais de risco gestacional, diversidade de participantes e uso de imagens realistas.

**Conclusão:**

Tecnologias mapeadas evidenciam avanços importantes, além da necessidade de desenvolver ferramentas mais específicas, inclusivas e validadas clinicamente. Tais recursos podem qualificar práticas pré-natais em Enfermagem e saúde e melhorar desfechos maternos e neonatais.

## INTRODUÇÃO

As Tecnologias Educativas em Saúde (TES) abrangem estratégias e recursos destinados à promoção do conhecimento em saúde para diversos públicos, visando facilitar a compreensão e promover o autocuidado^(1)^. No campo da obstetrícia, as TES vêm se consolidando como apoio relevante ao fortalecimento da saúde materno-infantil, especialmente no contexto do pré-natal, parto e pós-parto^(2)^.

Quanto aos temas abordados na área materno-infantil, estudos de revisão pontuam o predomínio de orientações sobre alimentação saudável, preparo para o parto, violência obstétrica, amamentação, cuidados puerperais e com o recém-nascido^(2, 3, 4)^, com limitada atenção às orientações sobre sinais de risco na gestação e ao início do trabalho de parto^(5)^. Tal lacuna é preocupante diante da persistência de desafios na saúde pública obstétrica brasileira, como a elevada mortalidade materna^(6)^.

Para uma busca oportuna por assistência, é fundamental que as gestantes reconheçam os sinais de trabalho de parto iminente ou em curso (contrações regulares, perda do tampão mucoso, rompimento da bolsa) e os sinais que indicam risco gestacional (sangramento vaginal, cefaleia intensa, visão turva, dor abdominal persistente, ausência de movimentos fetais, alterações no fluido vaginal, febre). No entanto, estudos indicam que apenas 39% das gestantes brasileiras conseguem identificar corretamente a maioria desses sinais, revelando necessidade urgente de estratégias educativas específicas no pré-natal^(5)^.

O desenvolvimento e a utilização de TES no contexto obstétrico alinham-se às diretrizes nacionais de fortalecimento da Rede de Atenção Materna e Infantil, como a Rede Cegonha e a Rede Alyne, ao ampliarem o acesso à informação qualificada e favorecerem a tomada de decisão segura pelas gestantes. No âmbito global, essas tecnologias também contribuem para o cumprimento do Objetivo de Desenvolvimento Sustentável (ODS), da Agenda 2030 das Nações Unidas, que visa assegurar uma vida saudável e promover o bem-estar para todos, em todas as idades^(7)^.

Antes da formulação desta revisão, foi realizada uma busca preliminar para verificar a existência de estudos de revisão e protocolos registrados que respondessem à pergunta da presente investigação. Essa busca foi conduzida nas plataformas PROSPERO, Open Science Framework (OSF), PubMed e Scopus. Foram encontradas revisões com escopos diferentes^(2, 3, 4)^, não contemplando especificamente TES sobre sinais de risco gestacional e trabalho de parto no contexto brasileiro, o que reforça a originalidade e relevância do estudo.

Assim, este estudo teve como objetivo: mapear tecnologias educativas sobre sinais de risco gestacional e/ou trabalho de parto desenvolvidas ou utilizadas para orientar gestantes no contexto do pré-natal brasileiro.

## MÉTODO

### Tipo de Estudo

Trata-se de uma revisão de escopo realizada em nove etapas, conforme o Manual do JBI (2024), e seguindo as diretrizes do Preferred Reporting Items for Systematic Reviews and Meta-Analyses extension for Scoping Reviews (PRISMA-ScR)^(8)^. O protocolo foi registrado na plataforma OSF (DOI: 10.17605/OSF.IO/24JW7).

### População e Critérios de Seleção

A pergunta de pesquisa foi estruturada com base na estratégia PCC, em que a população são as gestantes; o conceito, as TES voltadas à orientação sobre sinais de risco na gestação e sinais de trabalho de parto; e o contexto, o cuidado pré-natal brasileiro, independentemente do nível de atenção. Diante disso, definiu-se a pergunta: Quais tecnologias educativas têm sido desenvolvidas ou utilizadas no contexto do pré-natal brasileiro para orientar gestantes sobre sinais de risco gestacional e de trabalho de parto?

### Definição da Amostra

Foram incluídos estudos em português, inglês ou espanhol, com texto completo, sem restrição temporal, que abordassem TES sobre sinais de risco gestacional e/ou trabalho de parto no contexto do pré-natal brasileiro. Excluíram-se estudos que não contemplassem esses sinais, fossem voltados exclusivamente a outros públicos ou tratassem de revisões de qualquer natureza.

### Coleta de Dados

A estratégia de busca foi elaborada com base nas recomendações do *JBI* (2024) e construída pelas autoras, que possuem experiência prévia em revisões de escopo. Foram utilizados descritores controlados (DeCS e MeSH) e termos livres, devidamente adaptados à lógica de cada base. As buscas foram realizadas nas bases Scopus, Web of Science, PubMed/MEDLINE, LILACS, BDENF, BDTD e Google Scholar, com acesso às três primeiras por meio do Portal CAPES/CAFe. Nas bases internacionais, acrescentou-se o termo “Brazil” para delimitar o contexto nacional da investigação. As estratégias estão detalhadas no Quadro 1.

**Quadro 1:** Estratégias de busca utilizadas por base de dados – Fortaleza, Ceará, Brasil, 2025.

**LILACS e BDENF**
(“Educação em Saúde” OR “Tecnologia Educacional” OR “Material Educativo” OR “Recursos Educacionais”) AND (“Gravidez” OR “Gestantes” OR “Cuidado Pré-Natal”) AND (“Complicações na Gravidez” OR “Trabalho de Parto” OR “Parto”)
**Biblioteca Digital de Teses e Dissertações (BDTD)**
(“tecnologia educativa” OR “tecnologia educacional” OR “material educativo”) AND (“pré-natal” OR “gestação” OR “sinais de risco” OR “trabalho de parto” OR “parto”)
**Google Scholar**
(“tecnologia educativa” OR “tecnologia educacional” OR “material educativo” OR “recurso educativo” OR “estratégia educativa”) AND (“pré-natal” OR “gravidez” OR “gestantes”) AND (“sinais de risco” OR “complicações na gestação” OR “alertas na gravidez” OR “trabalho de parto” OR “sinais de parto”)
**PubMed/MEDLINE**
(“Health Education”[MeSH] OR “Educational Technology”[MeSH]) AND (“Pregnancy”[MeSH] OR “Prenatal Care”[MeSH] OR “Labor, Obstetric”[MeSH] OR “Pregnancy Complications”[MeSH]) AND (Brazil[MeSH])
**Scopus**
TITLE-ABS-KEY (“health education” OR “prenatal education” OR “educational technology”) AND (“pregnancy” OR “prenatal care” OR “labor” OR “childbirth” OR “pregnancy complications”) AND (“Brazil”)
**Web of Science**
TS = ((“health education” OR “prenatal education” OR “educational technology”) AND (“pregnancy” OR “prenatal care” OR “labor” OR “pregnancy complications”) AND (“Brazil”))

### Análise e Tratamento dos Dados

A triagem dos estudos foi realizada na plataforma Rayyan, em duas etapas: leitura de títulos/resumos e leitura completa, seguindo critérios definidos. Dois revisores atuaram de forma independente. Em caso de discordância, previa-se a atuação de um terceiro revisor, embora não tenha sido necessário. Após a triagem, os estudos incluídos foram submetidos à leitura na íntegra.

Os dados foram extraídos utilizando formulário elaborado pelos autores contendo informações como título, ano, unidade federativa da publicação, tipo de publicação, tipo e características da tecnologia, conteúdos abordados e forma de apresentação, tipos de imagens, público-alvo, estágio de validação e participantes envolvidos entre outros. Ressalta-se que as imagens foram classificadas por tipo - ilustrações e fotografias, e por finalidade- decorativas, sem relação direta com o conteúdo, e instrutivas, usadas para explicar ou exemplificar os sinais abordados^(9)^. O formulário foi previamente testado em três estudos para verificação da consistência e adequação dos campos.

A descrição dos resultados foi conduzida conforme as diretrizes metodológicas para revisões de escopo, respeitando a natureza exploratória e não avaliativa deste tipo de estudo^(8)^. Para embasar a discussão dos resultados, adotou-se o modelo PAGER (*Patterns, Advances, Gaps, Evidence for Practice, and Research Recommendations*), que orientou a análise em cinco dimensões: padrões identificados, avanços, lacunas, evidências para a prática e recomendações para pesquisas futuras^(10)^.

### Aspectos Éticos

Esta revisão de escopo utilizou exclusivamente dados secundários disponíveis publicamente e, por isso, não demandou aprovação por Comitê de Ética em Pesquisa. Todas as etapas foram conduzidas em conformidade com os princípios éticos de rigor metodológico, fidelidade na reprodução das informações e adequada atribuição das fontes.

## RESULTADOS

A busca em sete bases resultou na identificação de 1089 registros, dos quais, após triagem e análise de elegibilidade, 21 estudos foram incluídos. O diagrama de fluxo PRISMA-ScR, apresentado na Figura 1, ilustra detalhadamente este processo de seleção.

**Figura 1:** Fluxograma de triagem e inclusão dos estudos segundo PRISMA-ScR.

Fonte: Adaptado do Preferred Reporting Items for Systematic Reviews and Meta-Analyses Extension for Scoping Reviews, 2020.

A maioria dos estudos (62%) correspondeu a artigos publicados em periódicos nacionais de Enfermagem; os demais foram dissertações (28,5%) e teses (9,5%). A maior concentração de publicações ocorreu entre 2019 e 2022, com destaque para o ano de 2022. Quanto ao delineamento, 12 estudos eram metodológicos (57%), sete ensaios clínicos (33%), sendo quatro controlados e randomizados (19%) e três quase-experimentais de grupo único (14%), além de dois relatos de experiência (10%). Informações complementares estão disponíveis no Quadro 2.

**Quadro 2:** Caracterização dos estudos incluídos segundo ano, estado, formato de publicação, revista, tipo metodológico e autoria profissional – Fortaleza, Ceará, Brasil, 2025.

Título do estudo	Ano	Estado	Formato Revista	Tipo de estudo	Área de atuação dos autores
Cartilha educativa sobre trabalho de parto e parto: estudo de validade^(11)^	2024	MS	Artigo (Rev. Bras. Enferm)	Metodológico	Enfermagem
Conhecimento, atitude e prática sobre síndrome hipertensiva gestacional entre gestantes: ensaio clínico randomizado^(12)^	2022	CE	Artigo (Texto Contexto Enferm)	Ensaio clínico	Enfermagem
Validação de tecnologia educativa para mulheres indígenas sobre o pré-natal^(13)^	2022	CE	Dissertação	Metodológico	Enfermagem
Desenvolvimento do álbum seriado meu bebê vai nascer: guia da gestante para o trabalho de parto, parto e pós-parto^(14)^	2019	CE	Tese	Ensaio clínico	Enfermagem
Contribuição de gestantes na construção e avaliação de uma tecnologia educacional: o “Almanaque da Gestante”^(15)^	2021	ES	Artigo (Rev. Bras. Enferm)	Metodológico	Enfermagem Designer gráfico
Simplesmente mães: construção compartilhada de tecnologias sobre pré-natal de mulheres com deficiência visual^(16)^	2024	PA	Artigo (Cogitare Enferm)	Metodológico	Enfermagem
Quando ir para a maternidade? Educação em saúde sobre o trabalho de parto^(17)^	2017	MG	Artigo (Rev enfermUFPE)	Relato de experiência	Enfermagem Medicina
Infográfico para gestantes e acompanhantes: tecnologia educacional no contexto do atendimento obstétrico^(18)^	2023	SC	Artigo (Cogitare Enferm)	Metodológico	Enfermagem
Desenvolvimento de aplicativo móvel para o acompanhamento pré-natal e validação de conteúdo^(19)^	2022	RN	Artigo (Acta Paul Enferm)	Metodológico	Enfermagem
Eficácia de aplicativo móvel na adesão de gestantes às consultas de pré-natal: ensaio clínico randomizado^(20)^	2021	RN	Artigo (Rev. Bras. Enferm)	Ensaio clínico	Enfermagem
Intervenção Educativa no Cuidado Obstétrico através de um Aplicativo para Dispositivos Móveis: APP^(21)^	2018	MG	Artigo (Rev. Intern. em Líng. Portuguesa)	Relato de experiência	Medicina Eng.computação Análise de sistema
Intervenção educativa para conhecimento, atitude e prática de gestantes adolescentes no preparo para o parto vaginal: ensaio clínico randomizado^(22)^	2022	PE	Tese	Ensaio clínico	Enfermagem
SABER G-ESTAR: construção e validação de um aplicativo móvel para educação em saúde no ciclo gravídico-puerperal^(23)^	2021	RJ	Dissertação	Metodológico	Enfermagem
Desenvolvimento e validação de um protótipo de aplicativo para plataforma móvel para promoção da saúde de gestantes^(24)^	2019	CE	Dissertação	Metodológico	Enfermagem
Desenvolvimento de um protótipo de software para monitoramento de gestantes^(25)^	2019	AL	Dissertação	Metodológico	Enfermagem
Implementação de uma tecnologia de informação aplicada ao acompanhamento e promoção do cuidado à gestante^(26)^	2020	BA	Dissertação	Metodológico	Medicina
Desenvolvimento de website para gestantes: estudo metodológico^(27)^	2022	AC	Dissertação	Metodológico	Enfermagem
Avaliação de objeto virtual de aprendizagem sobre pré-natal para adolescentes grávidas na atenção básica^(28)^	2020	PI	Artigo (Acta Paul Enferm)	Metodológico	Enfermagem
Efeito de intervenção educativa online na qualidade de vida de gestantes adolescentes^(29)^	2022	PI	Artigo (Acta Paul Enferm)	Ensaio clínico	Enfermagem
Estratégia de comunicação e informação em saúde e percepção de sentir-se preparada para o parto: ensaio aleatorizado por conglomerados^(30)^	2019	SP	Artigo (Cad. Saúde Pública)	Ensaio clínico	Enfermagem Medicina Jornalismo Nutrição Psicologia Fisioterapia Biomedicina
Tecnologia educacional para primigestas: estudo quase experimental^(31)^	2022	RN	Artigo (Rev. esc. enferm. USP)	Ensaio clínico	Enfermagem

Esses estudos descreveram 18 tecnologias, desenvolvidas ou utilizadas no contexto do pré-natal brasileiro, entre 2017 e 2024. Ressalta-se que três dessas tecnologias (T9, T10 e T16) foram abordadas em dois estudos distintos, com enfoques complementares, como construção e validação em um, e avaliação de efetividade em outro.

Geograficamente, as tecnologias foram majoritariamente desenvolvidas no Nordeste (50%) e Sudeste (27,8%), com menor representação das regiões Norte, Sul e Centro-Oeste.

O Quadro 3 apresenta a síntese das tecnologias educativas mapeadas, organizadas conforme o formato (materiais impressos, recursos digitais e recursos audiovisuais), e contempla informações sobre os sinais abordados e a forma de apresentação utilizada em cada recurso.

**Quadro 3:** Organização das tecnologias educativas mapeadas quanto aos tipos, sinais de parto, sinais de risco e forma de apresentação – Fortaleza, Ceará, Brasil, 2025.

Tecnologia			Sinais de		Forma de apresentação
			Parto	Risco	
**Materiais Impressos (44%)**	T1	Cartilha Cada parto é uma história^(11)^	■	□	Texto, ilustrações decorativas e instrutivas, fotografias
	T2	Cartilha Prevenção da SHG^(12)^	□	■	Texto e ilustrações instrutivas
	T3	Cartilha Cuidados com a indígena grávida^(13)^	■	■	Texto e ilustrações decorativas
	T4	Álbum seriado Meu bebê vai nascer^(14)^	■	■	Texto e ilustrações instrutivas
	T5	Almanaque da gestante^(15)^	■	■	Histórias em quadrinhos, caça-palavras, cruzadinha
	T6	Guia Simplesmente Mães^(16)^	■	■	Texto adaptado para leitores de tela.
	T7	Folder Quando ir para maternidade?^(17)^	■	■	Texto, ilustrações decorativas
	T8	Infográfico obstétrico^(18)^	■	■	Textos em tópicos, símbolos e ilustrações instrutivas
**Recursos Digitais (50%)**	T9	Aplicativo Gestação Saudável^(19,20)^	■	■	Texto em menus interativos
	T10	Aplicativo Meu Pré-Natal^(21,22)^	■	■	Texto em perguntas e respostas, vídeos animados
	T11	Aplicativo Saber G-estar^(23)^	■	■	Texto, ilustrações instrutivas e decorativas
	T12	Aplicativo Gestar^(24)^	■	■	Texto e ilustrações instrutivas e decorativas
	T13	Aplicativo Mitera^(25)^	■	■	Questionário interativo, ícones
	T14	Webapp Juntos no pré-natal^(26)^	■	■	Texto em perguntas e respostas
	T15	Website Meu Parto^(27)^	■	□	Texto e ilustrações instrutivas
	T16	Objeto Virtual de Aprendizagem GESTAQ^(28,29)^	■	□	Texto, vídeo, fóruns interativos
	T17	Sistema de mensagens curtas de texto PRENACEL^(30)^	■	■	Mensagens de texto SMS
**Recursos Audiovisuais (6%)**	T18	Vídeo Quando ir para maternidade?^(31)^	■	■	Texto, áudio, ilustrações instrutivas e Libras

Legenda: ■ Sinal abordado; □ Sinal não abordado.

Em dez tecnologias (55,5%), os sinais de risco gestacional e/ou de trabalho de parto foram abordados de forma complementar, inseridos em propostas mais amplas de educação no ciclo gravídico-puerperal. Apenas cinco (27,8%) EHT concentraram-se exclusivamente na orientação sobre sinais de risco gestacional e/ou sinais de trabalho de parto.

A Figura 2 apresenta o diagrama de rede que sintetiza as conexões entre as tecnologias educativas incluídas e os conteúdos abordados. Observa-se a distribuição temática entre aquelas que tratam exclusivamente de sinais de risco gestacional, de sinais de trabalho de parto ou de ambos, permitindo visualizar a abrangência e o foco de cada tecnologia mapeada.

**Figura 2:** Diagrama de Rede das conexões entre tecnologias educativas, sinais de trabalho de parto e sinais de risco gestacional.

Quanto às validações a que as tecnologias mapeadas foram submetidas, estas são descritas no Quadro 4, contemplando a validação de qualidade, que inclui aspectos como conteúdo, aparência e usabilidade, e a validação clínica, voltada à análise dos efeitos no público-alvo. Os resultados referem-se à validação de qualidade, e os desfechos, à validação clínica.

**Quadro 4:** Síntese da validação, resultados e desfechos das tecnologias educativas mapeadas na revisão de escopo – Fortaleza, Ceará, Brasil, 2025.

Tecnologia	Tipo de validação	Resultados/Desfechos
T1^(11)^	Conteúdo e aparência	Alto IVC = 0,91 e boa consistência interna (alfa de Cronbach >0,8) na avaliação dos especialistas. Considerada compreensível e útil por 95,5% das gestantes.
T2^(12)^	Conteúdo, aparência e clínica	Validação por especialistas. Maiores informações não fornecidas. Melhorias significativas no CAP das gestantes sobre SHG (p < 0,05).
T3^(13)^	Conteúdo e aparência	Alcançou excelente confiabilidade e satisfação entre os especialistas (IVC = 0,95).
T4^(14)^	Conteúdo, aparência e clínica	Considerado de fácil compreensão pelas gestantes (IC = 99%) e alta aceitação pelos especialistas (IVC = 0,92, alfa de Cronbach = 0,9). Impacto positivo no conhecimento das gestantes sobre trabalho de parto, parto e pós-parto (p < 0,05).
T5^(15)^	Conteúdo e aparência	Considerado pelas gestantes de fácil entendimento, autoexplicativo, esteticamente atrativo e motivador de boas práticas (IC = 92,1%).
T6^(16)^	Qualitativa por entrevista	Avaliado positivamente pelo público-alvo, por atender demandas específicas e promover autonomia.
T7^(17)^	Conteúdo	Adaptado por especialistas e distribuído nas consultas para informar sobre sinais de alerta e parto.
T8^(18)^	Conteúdo e aparência	Considerado adequado para disseminação de informações por especialistas; IVC de 83,3% após duas rodadas Delphi.
T9^(19,20)^	Conteúdo, usabilidade e clínica	Considerado válido por enfermeiros e profissionais de informática com nível de consenso de 83,3% após duas rodadas Delphi. Alto índice de adesão ao pré-natal entre gestantes do grupo intervenção (83,3% vs. 7,7% no grupo controle; p < 0,05).
T10^(21,22)^	Conteúdo, usabilidade e clínica	Avaliado positivamente por especialistas e gestantes. Favoreceu o CAP de adolescentes sobre o preparo para o parto.
T11^(23)^	Usabilidade e aceitação	Significativo nível de satisfação entre as gestantes (IVC = 0,98 e Kappa = 0,96)
T12^(24)^	Conteúdo, usabilidade e aparência	Protótipo bem aceito entre os especialistas, IVC: conteúdo (0,68), usabilidade (0,62), aparência (0,93).
T13^(25)^	Usabilidade e aparência	Fácil manuseio para 87,5% das gestantes; favoreceu a percepção de sintomas de risco.
T14^(26)^	Qualitativa por entrevista	Bem aceito pelas usuárias, especialmente primigestas
T15^(27)^	Conteúdo, aparência e usabilidade	Concordância superior a 0,8 entre especialistas e gestantes nas avaliações de conteúdo, aparência e usabilidade.
T16^(28,29)^	Conteúdo, usabilidade, funcionalidade	Avaliações ≥ 80% nas dimensões de qualidade, funcionalidade e eficiência pelos especialistas. Impacto positivo na qualidade de vida das gestantes adolescentes (p < 0,001).
T17^(30)^	Conteúdo e clínica	Validação por gestantes e especialistas. Maiores informações não fornecidas. Uso associado à maior percepção de preparo para o parto.
T18^(31)^	Conteúdo e clínica	Alta satisfação entre primigestas (média de 9,92) Aumento significativo no conhecimento sobre sinais clínicos após a intervenção

Legenda**:** IVC = Índice de Validação do Conteúdo; IC = Índice de Concordância; CAP = Conhecimento Atitude Prática.

## DISCUSSÃO

A análise das 18 TES mapeadas revelou predominância dos recursos digitais, refletindo tendências contemporâneas no campo da educação em saúde, com destaque para maior acessibilidade, alcance da informação e possibilidade de atualização ágil de conteúdos^(23,24,31)^.

### Padrões Identificados (Patterns)

Observou-se um padrão de linguagem clara, acessível, simples e culturalmente adequado, aliado ao uso de ilustrações instrutivas para explicar ou exemplificar os sinais de risco e aspectos do trabalho de parto. Algumas TES utilizaram estratégias lúdicas como jogos e histórias em quadrinhos^(15)^, narração de histórias^(14,28,31)^, fóruns e canais de comunicação com profissionais de saúde, promovendo engajamento e fortalecendo o vínculo entre gestantes e equipes de assistência^(19,28,30)^.

### Avanços Observados (Advances)

Avanços foram evidenciados no rigor metodológico adotado na construção das TES, com forte embasamento em referenciais teóricos atualizados e validação por especialistas e/ou gestantes. Destacam-se tecnologias desenvolvidas a partir das necessidades do público-alvo^(13,15,16,18,19,23^, fator crucial para aumentar a aceitabilidade e a efetividade.

Além disso, a realização de validações clínicas reforçou a credibilidade de algumas tecnologias, apontando resultados positivos, como o aumento do conhecimento sobre sinais de risco e trabalho de parto^(14,31)^, maior percepção de preparo para o parto^(30)^ e melhorias significativas no CAP sobre a SHG^(12)^ e o parto^(22)^, fortalecendo a credibilidade e favorecendo a replicabilidade dessas tecnologias.

### Lacunas Identificadas (Gaps)

Apesar dos avanços, foram identificadas lacunas relevantes. Poucas tecnologias abordaram exclusivamente os sinais de risco gestacional ou trabalho de parto, sendo comum a inserção desses conteúdos de forma secundária em propostas educativas mais amplas, o que pode comprometer sua eficácia em situações que exigem tomada de decisão rápida^(12,17,18,25,31)^.

Identificou-se também escassez no uso de recursos visuais realistas, como fotografias, potencialmente mais eficazes na representação de sinais clínicos^(11)^. Além disso, a produção científica apresentou desigualdade geográfica, concentrando-se majoritariamente nas regiões Nordeste^(12, 13, 14,19, 20, 21,24, 25, 26,28,29,31)^ e Sudeste^(15,17,21,23,30)^, com pouca representatividade de regiões como o Norte, marcado por maiores desafios de acesso, diversidade cultural e piores indicadores obstétricos^(27)^.

Outro aspecto crítico foi a limitação na representatividade dos participantes nos processos de construção e validação. A participação concentrou-se nas regiões onde os estudos foram conduzidos, o que restringe a diversidade cultural e linguística. Alguns autores relataram dificuldade para reunir juízes de diferentes estados^(11)^ e baixa adesão dos avaliadores convidados^(19,27)^. Além disso, a inclusão de gestantes adolescentes (10 a 19 anos) foi pouco explorada, apesar da maior vulnerabilidade à desinformação e insegurança frente à gestação e ao parto^(13,14,22)^.

### Implicações Para a Prática (Evidence for Practice)

Os achados desta revisão oferecem subsídios relevantes para o aprimoramento de políticas públicas voltadas à saúde materno-infantil, especialmente no âmbito do Sistema Único de Saúde (SUS). As TES mapeadas apresentaram evidências promissoras para qualificar o cuidado pré-natal, promover o reconhecimento precoce de sinais críticos e fortalecer a autonomia das gestantes, de modo que seu uso complementar à atuação profissional pode contribuir para a redução de desfechos maternos e neonatais adversos.

Assim, a incorporação dessas tecnologias às ações educativas do pré-natal, aos protocolos assistenciais e às estratégias da Rede Cegonha e da Rede Alyne configura uma oportunidade em potencial para promover uma atenção mais responsiva, inclusiva e baseada em evidências.

O envolvimento ativo da Enfermagem no desenvolvimento e aplicação dessas tecnologias reforça o papel estratégico da profissão na promoção da saúde materno-infantil e na inovação educacional^(11, 12, 13, 14, 15, 16, 17, 18, 19, 20,22, 23, , 24, 25,27, 28, 29, 30, 31)^. Iniciativas que incorporam acessibilidade, como o guia voltado a mulheres com deficiência visual^(16)^ e o vídeo com tradução em Libras^(31)^, merecem destaque como práticas que promovem equidade no acesso à informação.

### Recomendações Para Pesquisas Futuras (Research Recommendations)

Recomenda-se o desenvolvimento de novas tecnologias que ampliem o uso de recursos visuais realistas, como fotografias, associadas a ilustrações explicativas, respeitando o contexto sociocultural e a sensibilidade do tema. Tal combinação pode potencializar a compreensão dos sinais clínicos, sobretudo em públicos com diferentes níveis de letramento em saúde^(9, 10, 11,22)^.

Para superar as limitações geográficas identificadas, é importante descentralizar os processos de produção e validação das tecnologias, e envolver participantes de diferentes regiões do país. A inclusão de realidades diversas pode ampliar a aplicabilidade e a sensibilidade cultural das ferramentas educativas, contribuindo para sua efetividade em múltiplos contextos. Recomenda-se, para isso, a formação de parcerias entre instituições de pesquisa de distintas regiões, em caráter colaborativo ou multicêntrico, favorecendo a diversidade amostral e o compartilhamento metodológico. Estratégias complementares para captação de participantes podem incluir o uso de redes sociais, divulgação por associações científicas e profissionais, ampliação dos canais de contato (e-mail, WhatsApp, ligações telefônicas), envio de lembretes automatizados e validação da viabilidade dos prazos instituídos para devolutivas, de modo a favorecer o engajamento, a corresponsabilização e a retenção dos participantes ao longo do processo.

No caso das adolescentes, a inclusão ativa como participantes nos processos de construção e avaliação das tecnologias deve ser estimulada^(22,28,29)^. Sugere-se, para isso, a articulação com escolas, unidades básicas de saúde e serviços especializados em saúde sexual e reprodutiva, o que pode facilitar o recrutamento, o engajamento e a escuta qualificada desse grupo.

Por fim, reforça-se a importância da realização de validações clínicas rigorosas, com delineamentos robustos e avaliação de desfechos clínicos e comportamentais, como conhecimento, atitude, prática e adesão ao pré-natal. Essas resultados são fundamentais para respaldar a adoção das tecnologias no cuidado pré-natal e sua incorporação em políticas públicas baseadas em evidências^(12,14,20,22,29)^.

### Limitações do Estudo

Embora a BDTD tenha sido utilizada como fonte de literatura cinzenta, a não inclusão sistemática de outros repositórios institucionais não indexados pode ter limitado a identificação de tecnologias educativas. Além disso, embora as estratégias de busca tenham sido amplas e conduzidas com rigor, é possível que a seleção de bases e descritores tenha limitado a identificação de estudos relevantes.

## CONCLUSÃO

Esta revisão de escopo mapeou 18 tecnologias educativas desenvolvidas ou utilizadas no contexto do pré-natal brasileiro para orientar gestantes sobre sinais de risco gestacional e sinais indicativos do trabalho de parto.

Os resultados evidenciam avanços na incorporação de recursos digitais, linguagem acessível e validações rigorosas, com potencial para qualificar o cuidado pré-natal e fortalecer a autonomia das gestantes. No entanto, lacunas temáticas, metodológicas e geográficas persistem, incluindo a limitada abordagem exclusiva dos sinais críticos, a baixa representatividade regional e a escassa inclusão de gestantes adolescentes.

Os achados contribuem para a ciência da Enfermagem ao indicar caminhos para o desenvolvimento de tecnologias mais específicas, inclusivas e validadas clinicamente, capazes de apoiar práticas assistenciais baseadas em evidências e impactar positivamente os desfechos maternos e neonatais.

Reforça-se a necessidade de futuras pesquisas focadas na ampliação da diversidade cultural dos públicos envolvidos e na utilização de recursos visuais mais realistas para potencializar a efetividade das intervenções educativas.

## Data Availability

Todo o conjunto de dados que dá suporte aos resultados deste estudo está disponível mediante solicitação ao autor correspondente.

# Translation: Tecnologías educativas sobre señales de riesgo gestacional y/o parto para embarazadas: revisión de alcance*

**DOI:** 10.1590/1980-220X-REEUSP-2025-0188es

**Authors:** Thaís Jormanna Pereira Silva, Ana Carolina Nunes de Macêdo, Caroline Magalhães de Alcântara, Maria Veraci Oliveira Queiroz, Igor Cordeiro Mendes, Thaís Marques Lima, Juliana Alencar Moreira Borges

**Affiliations:** 1 Universidade Estadual do Ceará, Programa de Pós-Graduação em Cuidados Clínicos em Enfermagem e Saúde, Fortaleza, CE, Brasil.; 2 Universidade Federal do Rio Grande do Norte, Faculdade de Ciências da Saúde do Trairi, Santa Cruz, RN, Brasil.

**Keywords:** Tecnología Educacional, Atención Prenatal, Educación en Salud, Complicaciones del Embarazo, Trabajo de Parto

## Abstract

**Objetivo:**

Mapear tecnologías educativas sobre señales de riesgo gestacional y/o de parto desarrolladas o utilizadas para orientar a las embarazadas en el contexto de la atención prenatal brasileña.

**Método:**

Revisión del alcance realizada de acuerdo con JBI con búsquedas realizadas en las siguientes bases de datos: Scopus, Web of Science, PubMed/MEDLINE, LILACS, BDENF, BDTD y Google Scholar, sin restricción de tiempo. Selección y extracción de datos en etapas independientes por pares, y síntesis narrativa según el modelo PAGER.

**Resultados:**

Se incluyeron 21 estudios que describieron 18 tecnologías: 10 digitales (55,5%), 8 impresas (44%) y 1 audiovisual (5,5%). La mayoría (n = 13; 72,2%) abordó los signos de forma complementaria. Predominaron recursos con lenguaje accesible, elementos instructivos y la implicación de Enfermería en su diseño. Se identificaron brechas temáticas, metodológicas y geográficas, especialmente en el enfoque exclusivo en signos de riesgo gestacional, diversidad de participantes y uso de imágenes realistas.

**Conclusión:**

Las tecnologías mapeadas resaltan avances importantes, además de la necesidad de desarrollar herramientas más específicas, inclusivas y clínicamente validadas. Estos recursos pueden calificar las prácticas prenatales en enfermería y salud y mejorar los resultados maternos y neonatales.

## INTRODUCCIÓN

Las Tecnologías Educativas en Salud (TES) abarcan estrategias y recursos destinados a promover el conocimiento en salud para diversos públicos, con el fin de facilitar la comprensión y promover el autocuidado^(1)^. En el campo de la obstetricia, las TES se han consolidado como un apoyo relevante para el fortalecimiento de la salud materno-infantil, especialmente en el contexto del prenatal, el parto y el posparto^(2)^.

En cuanto a los temas abordados en el área materno-infantil, los estudios de revisión señalan el predominio de las orientaciones sobre alimentación saludable, preparación para el parto, violencia obstétrica, lactancia materna, cuidados puerperales y cuidados del recién nacido^(2, 3, 4)^, con una atención limitada a las orientaciones sobre los signos de riesgo en el embarazo y el inicio del trabajo de parto^(5)^. Esta brecha de conocimiento es preocupante ante la persistencia de retos en la salud pública obstétrica brasileña, como la elevada mortalidad materna^(6)^.

Para buscar asistencia oportuna, es fundamental que las embarazadas reconozcan los signos de parto inminente o en curso (contracciones regulares, pérdida del tapón mucoso, rotura de la bolsa) y los signos que indican riesgo gestacional (sangrado vaginal, cefalea intensa, visión borrosa, dolor abdominal persistente, ausencia de movimientos fetales, alteraciones en el flujo vaginal, fiebre). Sin embargo, los estudios indican que solo el 39 % de las mujeres embarazadas brasileñas pueden identificar correctamente la mayoría de estos signos, lo que revela la necesidad urgente de estrategias educativas específicas en el ámbito prenatal^(5)^.

El desarrollo y el uso de las TES en el contexto obstétrico se alinean con las directrices nacionales para fortalecer la Red de Atención Materno-Infantil, como la Red Cegonha (Cigüeña) y la Red Alyne, al ampliar el acceso a información cualificada y favorecer la toma de decisiones seguras por parte de las embarazadas. A nivel mundial, estas tecnologías también contribuyen al cumplimiento del Objetivo de Desarrollo Sostenible (ODS) de la Agenda 2030 de las Naciones Unidas, que tiene por objeto garantizar una vida sana y promover el bienestar para todos, en todas las edades^(7)^.

Antes de formular esta revisión, se realizó una búsqueda preliminar para verificar la existencia de estudios de revisión y protocolos registrados que respondieran a la pregunta de la presente investigación. Esta búsqueda se llevó a cabo en las plataformas PROSPERO, Open Science Framework (OSF), PubMed y Scopus. Se encontraron revisiones con diferentes alcances^(2, 3, 4)^, que no contemplaban específicamente las TES sobre señales de riesgo gestacional y trabajo de parto en el contexto brasileño, lo que refuerza la originalidad y relevancia del estudio.

Así, este estudio tuvo como objetivo: mapear las tecnologías educativas sobre señales de riesgo gestacional y/o trabajo de parto desarrolladas o utilizadas para orientar a las gestantes en el contexto de la atención prenatal brasileña.

## MÉTODO

### Tipo de Estudio

Se trata de una revisión de alcance realizada en nueve etapas, de acuerdo con el Manual del JBI (2024) y siguiendo las directrices de los Elementos de información preferidos para las revisiones sistemáticas y los metaanálisis, extensión para revisiones de alcance (PRISMA-ScR)(8). El protocolo se registró en la plataforma OSF (DOI: 10.17605/OSF.IO/24JW7).

### Población y Criterios de Selección

La pregunta de investigación se estructuró basándose en la estrategia PCC, en la que la población son las mujeres embarazadas; el concepto, las TES orientadas a la orientación sobre los signos de riesgo en el embarazo y los signos de parto; y el contexto, la atención prenatal brasileña, independientemente del nivel de atención. En vista de ello, se definió la pregunta: ¿Qué tecnologías educativas se han desarrollado o utilizado en el contexto de la atención prenatal brasileña para orientar a las mujeres embarazadas sobre los signos de riesgo gestacional y de parto?

### Definición de la Muestra

Se incluyeron estudios en portugués, inglés o español, con texto completo, sin restricción temporal, que abordaran las TES sobre señales de riesgo gestacional y/o parto en el contexto de la atención prenatal brasileña. Se excluyeron los estudios que no contemplaban estas señales, que estaban dirigidos exclusivamente a otros públicos o que trataban de revisiones de cualquier tipo.

### Recolección de Datos

La estrategia de búsqueda se elaboró basándose en las recomendaciones de la *JBI* (2024) y fue construida por las autoras, que cuentan con experiencia previa en revisiones de alcance. Se utilizaron descriptores controlados (DeCS y MeSH) y términos libres, debidamente adaptados a la lógica de cada base. Las búsquedas se realizaron en las bases Scopus, Web of Science, PubMed/MEDLINE, LILACS, BDENF, BDTD y Google Scholar, con acceso a las tres primeras a través del Portal CAPES/CAFe. En las bases internacionales, se añadió el término «Brasil» para delimitar el contexto nacional de la investigación. Las estrategias se detallan en la Cuadro 1.

**Cuadro 1:** Estrategias de búsqueda utilizadas por base de datos – Fortaleza, Ceará, Brasil, 2025.

**LILACS y BDENF**
(“Educação em Saúde” OR “Tecnologia Educacional” OR “Material Educativo” OR “Recursos Educacionais”) AND (“Gravidez” OR “Gestantes” OR “Cuidado Pré-Natal”) AND (“Complicações na Gravidez” OR “Trabalho de Parto” OR “Parto”)
**Biblioteca Digital de Tesis y Disertaciones (BDTD)**
(“tecnologia educativa” OR “tecnologia educacional” OR “material educativo”) AND (“pré-natal” OR “gestação” OR “sinais de risco” OR “trabalho de parto” OR “parto”)
**Google Scholar**
(“tecnologia educativa” OR “tecnologia educacional” OR “material educativo” OR “recurso educativo” OR “estratégia educativa”) AND (“pré-natal” OR “gravidez” OR “gestantes”) AND (“sinais de risco” OR “complicações na gestação” OR “alertas na gravidez” OR “trabalho de parto” OR “sinais de parto”)
**PubMed/MEDLINE**
(“Health Education”[MeSH] OR “Educational Technology”[MeSH]) AND (“Pregnancy”[MeSH] OR “Prenatal Care”[MeSH] OR “Labor, Obstetric”[MeSH] OR “Pregnancy Complications”[MeSH]) AND (Brazil[MeSH])
**Scopus**
TITLE-ABS-KEY (“health education” OR “prenatal education” OR “educational technology”) AND (“pregnancy” OR “prenatal care” OR “labor” OR “childbirth” OR “pregnancy complications”) AND (“Brazil”)
**Web of Science**
TS = ((“health education” OR “prenatal education” OR “educational technology”) AND (“pregnancy” OR “prenatal care” OR “labor” OR “pregnancy complications”) AND (“Brazil”))

### Análisis y Tratamiento de los Datos

La selección de los estudios se realizó en la plataforma Rayyan, en dos etapas: lectura de títulos/resúmenes y lectura completa, siguiendo criterios definidos. Dos revisores trabajaron de forma independiente. En caso de desacuerdo, se preveía la intervención de un tercer revisor, aunque no fue necesario. Tras la selección, los estudios incluidos se sometieron a una lectura completa.

Los datos se extrajeron utilizando un formulario elaborado por los autores que contenía información como el título, el año, la unidad federativa de la publicación, el tipo de publicación, el tipo y las características de la tecnología, los contenidos abordados y la forma de presentación, los tipos de imágenes, el público destinatario, la fase de validación y los participantes involucrados, entre otros. Cabe destacar que las imágenes se clasificaron por tipo (ilustraciones y fotografías) y por finalidad (decorativas, sin relación directa con el contenido, e instructivas, utilizadas para explicar o ejemplificar los signos abordados^(9)^). El formulario se probó previamente en tres estudios para verificar la coherencia y la adecuación de los campos.

La descripción de los resultados se llevó a cabo de acuerdo con las directrices metodológicas para revisiones de alcance, respetando la naturaleza exploratoria y no evaluativa de este tipo de estudio^(8)^. Para fundamentar la discusión de los resultados, se adoptó el modelo PAGER (*Patterns, Advances, Gaps, Evidence for Practice, and Research Recommendations - Patrones, Avances, Brechas, Evidencia para la Práctica y Recomendaciones para la Investigación*), que orientó el análisis en cinco dimensiones: patrones identificados, avances, lagunas, evidencia para la práctica y recomendaciones para futuras investigaciones^(10)^.

### Aspectos Éticos

Esta revisión del alcance utilizó exclusivamente datos secundarios disponibles públicamente y, por lo tanto, no requirió la aprobación de un Comité de Ética en Investigación. Todas las etapas se llevaron a cabo de conformidad con los principios éticos de rigor metodológico, fidelidad en la reproducción de la información y adecuada atribución de las fuentes.

## RESULTADOS

La búsqueda en siete bases de datos dio como resultado la identificación de 1089 registros, de los cuales, tras la selección y el análisis de elegibilidad, se incluyeron 21 estudios. El diagrama de flujo PRISMA-ScR, presentado en la Figura 1, ilustra en detalle este proceso de selección.

**Figura 1:** Flujograma de selección e inclusión de los estudios según PRISMA-ScR.

Fuente: Adaptado de Preferred Reporting Items for Systematic Reviews and Meta-Analyses Extension for Scoping Reviews, 2020.

La mayoría de los estudios (62 %) correspondieron a artículos publicados en revistas nacionales de enfermería; el resto fueron disertaciones (28,5 %) y tesis (9,5 %). La mayor concentración de publicaciones se produjo entre 2019 y 2022, con especial destaque para el año 2022. En cuanto al diseño, 12 estudios eran metodológicos (57 %), siete ensayos clínicos (33 %), cuatro de ellos controlados y aleatorios (19 %) y tres cuasi-experimentales de grupo único (14 %), además de dos informes de experiencia (10 %). En el Cuadro 2 se ofrece información complementaria.

**Cuadro 2:** Caracterización de los estudios incluidos según año, estado, formato de publicación, revista, tipo metodológico y autoría profesional – Fortaleza, Ceará, Brasil, 2025.

Título del estudio	Año	Estado	Formato Revista	Tipo de estudio	Área de actuación de los autores
Cartilha educativa sobre trabalho de parto e parto: estudo de validade^(11)^	2024	MS	Artículo (Rev. Bras. Enferm)	Metodológico	Enfermería
Conhecimento, atitude e prática sobre síndrome hipertensiva gestacional entre gestantes: ensaio clínico randomizado^(12)^	2022	CE	Artículo (Texto Contexto Enferm)	Ensayo clínico	Enfermería
Validação de tecnologia educativa para mulheres indígenas sobre o pré-natal^(13)^	2022	CE	Disertación	Metodológico	Enfermería
Desenvolvimento do álbum seriado meu bebê vai nascer: guia da gestante para o trabalho de parto, parto e pós-parto^(14)^	2019	CE	Tesis	Ensayo clínico	Enfermería
Contribuição de gestantes na construção e avaliação de uma tecnologia educacional: o “Almanaque da Gestante”^(15)^	2021	ES	Artículo (Rev. Bras. Enferm)	Metodológico	Enfermería Designer gráfico
Simplesmente mães: construção compartilhada de tecnologias sobre pré-natal de mulheres com deficiência visual^(16)^	2024	PA	Artículo (Cogitare Enferm)	Metodológico	Enfermería
Quando ir para a maternidade? Educação em saúde sobre o trabalho de parto^(17)^	2017	MG	Artículo (Rev enfermUFPE)	Relato de experiencia	Enfermería Medicina
Infográfico para gestantes e acompanhantes: tecnologia educacional no contexto do atendimento obstétrico^(18)^	2023	SC	Artículo (Cogitare Enferm)	Metodológico	Enfermería
Desenvolvimento de aplicativo móvel para o acompanhamento pré-natal e validação de conteúdo^(19)^	2022	RN	Artículo (Acta Paul Enferm)	Metodológico	Enfermería
Eficácia de aplicativo móvel na adesão de gestantes às consultas de pré-natal: ensaio clínico randomizado^(20)^	2021	RN	Artículo (Rev. Bras. Enferm)	Ensaio clínico	Enfermería
Intervenção Educativa no Cuidado Obstétrico através de um Aplicativo para Dispositivos Móveis: APP^(21)^	2018	MG	Artículo (Rev. Intern. em Líng. Portuguesa)	Relato de experiencia	Medicina Ing.computación Análise de sistema
Intervenção educativa para conhecimento, atitude e prática de gestantes adolescentes no preparo para o parto vaginal: ensaio clínico randomizado^(22)^	2022	PE	Tese	Ensaio clínico	Enfermería
SABER G-ESTAR: construção e validação de um aplicativo móvel para educação em saúde no ciclo gravídico-puerperal^(23)^	2021	RJ	Disertación	Metodológico	Enfermería
Desenvolvimento e validação de um protótipo de aplicativo para plataforma móvel para promoção da saúde de gestantes^(24)^	2019	CE	Disertación	Metodológico	Enfermería
Desenvolvimento de um protótipo de software para monitoramento de gestantes^(25)^	2019	AL	Disertación	Metodológico	Enfermería
Implementação de uma tecnologia de informação aplicada ao acompanhamento e promoção do cuidado à gestante^(26)^	2020	BA	Disertación	Metodológico	Medicina
Desenvolvimento de website para gestantes: estudo metodológico^(27)^	2022	AC	Disertación	Metodológico	Enfermería
Avaliação de objeto virtual de aprendizagem sobre pré-natal para adolescentes grávidas na atenção básica^(28)^	2020	PI	Artículo (Acta Paul Enferm)	Metodológico	Enfermería
Efeito de intervenção educativa online na qualidade de vida de gestantes adolescentes^(29)^	2022	PI	Artículo (Acta Paul Enferm)	Ensayo clínico	Enfermería
Estratégia de comunicação e informação em saúde e percepção de sentir-se preparada para o parto: ensaio aleatorizado por conglomerados^(30)^	2019	SP	Artículo (Cad. Saúde Pública)	Ensayo clínico	Enfermería Medicina Periodismo Nutrición Psicología Fisioterapia Biomedicina
Tecnologia educacional para primigestas: estudo quase experimental^(31)^	2022	RN	Artículo (Rev. esc. enferm. USP)	Ensayo clínico	Enfermería

Estos estudios describieron 18 tecnologías, desarrolladas o utilizadas en el contexto de la atención prenatal brasileña, entre 2017 y 2024. Cabe destacar que tres de estas tecnologías (T9, T10 y T16) fueron abordadas en dos estudios distintos, con enfoques complementarios, como la construcción y validación en uno, y la evaluación de la eficacia en otro.

Geográficamente, las tecnologías se desarrollaron principalmente en el Nordeste (50 %) y el Sudeste (27,8 %), con una menor representación de las regiones Norte, Sur y Centro-Oeste.

La Cuadro 3 resume las tecnologías educativas mapeadas, organizadas según el formato (materiales impresos, recursos digitales y recursos audiovisuales), e incluye información sobre los signos cubiertos y la forma de presentación utilizada en cada recurso.

**Cuadro 3:** Organización de las tecnologías educativas mapeadas en términos de tipo, signos del parto, signos de riesgo y forma de presentación – Fortaleza, Ceará, Brasil, 2025.

Tecnología			Signos de		Forma de presentación
			Parto	Riesgo	
**Material impreso (44%)**	T1	Folleto Cada nacimiento es una historia^(11)^	■	□	Texto, ilustraciones decorativas e instructivas, fotografías
	T2	Folleto de prevención SHG^(12)^	□	■	Texto e ilustraciones instructivas
	T3	Folleto de atención a la mujer indígena embarazada^(13)^	■	■	Texto e ilustraciones decorativas
	T4	Serie de álbumes Mi bebé está a punto de nacer^(14)^	■	■	Texto e ilustraciones instructivas
	T5	Almanaque para embarazadas^(15)^	■	■	Cómics, sopas de letras, crucigramas
	T6	Guía Simplemente Mamás^(16)^	■	■	Texto adaptado para lectores de pantalla.
	T7	¿Cuándo ir a la maternidad? carpeta^(17)^	■	■	Texto, ilustraciones decorativas
	T8	Infografía obstétrica^(18)^	■	■	Texto temático, símbolos e ilustraciones instructivas
**Recursos digitales (50%)**	T9	App Embarazo Saludable^(19,20)^	■	■	Texto en menús interactivos
	T10	Mi aplicación de cuidados prenatales^(21,22)^	■	■	Texto en preguntas y respuestas, vídeos animados
	T11	Aplicación Saber G-estar^(23)^	■	■	Texto, ilustraciones instructivas y decorativas
	T12	Aplicación Gestar^(24)^	■	■	Texto e ilustraciones instructivas y decorativas
	T13	Aplicación Mitera^(25)^	■	■	Test interactivo, iconos
	T14	Webapp prenatal Juntos^(26)^	■	■	Texto en preguntas y respuestas
	T15	Sitio web Mi nacimiento^(27)^	■	□	Texto instructivo e ilustraciones
	T16	Objeto virtual de aprendizaje GESTAQ^(28,29)^	■	□	Texto, vídeo, foros interactivos
	T17	Sistema de mensajes cortos de texto PRENACEL^(30)^	■	■	Mensajes de texto SMS
**Recursos audiovisuales (6%)**	T18	Vídeo ¿Cuándo ir a la maternidad?^(31)^	■	■	Texto, audio, ilustraciones didácticas y Libras

Subtítulos: ■ Signo abordado; □ Signo no abordado.

En diez tecnologías (55,5%), los signos de riesgo gestacional y/o parto se abordaron de forma complementaria, como parte de propuestas educativas más amplias en el ciclo embarazo-puerperio. Sólo cinco (27,8%) TSE se centraron exclusivamente en la orientación sobre los signos de riesgo gestacional y/o los signos del parto.

La Figura 2 muestra el diagrama de red que resume las conexiones entre las tecnologías educativas incluidas y los contenidos tratados. Podemos ver la distribución temática entre las que tratan exclusivamente los signos de riesgo gestacional, los signos del parto o ambos, lo que nos permite visualizar alcance y el enfoque de cada tecnología mapeada.

**Figura 2:** Diagrama de red de las conexiones entre tecnologías educativas, signos de trabajo de parto y signos de riesgo gestacional

En cuanto a las validaciones a las que se sometieron las tecnologías mapeadas, éstas se describen en la Cuadro 4, abarcando la validación de calidad, que incluye aspectos como el contenido, la apariencia y la usabilidad, y la validación clínica, dirigida a analizar los efectos sobre el público objetivo. Los resultados se refieren a la validación cualitativa y los resultados a la validación clínica.

**Cuadro 4:** Resumen de la validación, los resultados y las consecuencias de las tecnologías educativas identificadas en la revisión del alcance – Fortaleza, Ceará, Brasil, 2025.

Tecnología	Tipo de validación	Resultados
T1^(11)^	Contenido y apariencia	CVI = 0,91 alto y buena consistencia interna (alfa de Cronbach >0,8) en la evaluación de los expertos. Considerado comprensible y útil por el 95,5% de las embarazadas.
T2^(12)^	Contenido, aspecto y clínica	Validado por expertos. No se facilitó más información. Mejoras significativas en el CAP de las embarazadas sobre SHG (p < 0,05).
T3^(13)^	Contenido y apariencia	Obtuvo una excelente fiabilidad y satisfacción entre los expertos (CVI = 0,95).
T4^(14)^	Contenido, aspecto y práctica clínica	Considerado fácil de entender por las embarazadas (IC = 99%) y altamente aceptado por los expertos (CVI = 0,92, alfa de Cronbach = 0,9). Impacto positivo en los conocimientos de las embarazadas sobre el trabajo de parto, el parto y el posparto (p < 0,05).
T5^(15)^	Contenido y aspecto	Considerado por las gestantes como fácil de entender, autoexplicativo, estéticamente atractivo y motivador de buenas prácticas (IC = 92,1%).
T6^(16)^	Entrevista cualitativa	Evaluado positivamente por el público objetivo por satisfacer demandas específicas y promover la autonomía.
T7^(17)^	Contenido	Adaptado por especialistas y distribuido en las consultas para informar sobre los signos de alarma y el parto.
T8^(18)^	Contenido y aspecto	Considerado adecuado para la difusión de información por los especialistas; IVC del 83,3% tras dos rondas Delphi.
T9^(19,20)^	Contenido, facilidad de uso y clínica	Considerado válido por las enfermeras y los informáticos con un nivel de consenso del 83,3% tras dos rondas Delphi. Alta tasa de adherencia a los cuidados prenatales entre las embarazadas del grupo de intervención (83,3% frente a 7,7% en el grupo de control; p < 0,05).
T10^(21,22)^	Contenido, usabilidad y práctica clínica	Evaluado positivamente por especialistas y gestantes. Favorecido PAC de adolescentes sobre preparación al parto.
T11^(23)^	Usabilidad y aceptación	Nivel de satisfacción significativo entre las embarazadas (CVI = 0,98 y Kappa = 0,96).
T12^(24)^	Contenido, usabilidad y aspecto	Prototipo bien aceptado por los expertos, CVI: contenido (0,68), usabilidad (0,62), aspecto (0,93).
T13^(25)^	Usabilidad y aspecto	Fácil de usar para el 87,5% de las embarazadas; favoreció la percepción de los síntomas de riesgo.
T14^(26)^	Entrevista cualitativa	Bien aceptado por las usuarias, especialmente las primigrávidas
T15^(27)^	Contenido, apariencia y facilidad de uso	Acuerdo superior a 0,8 entre expertos y embarazadas en las evaluaciones de contenido, apariencia y usabilidad.
T16^(28,29)^	Contenido, usabilidad y funcionalidad	Evaluaciones ≥ 80% en las dimensiones de calidad, funcionalidad y eficiencia por parte de los expertos. Impacto positivo en la calidad de vida de las adolescentes embarazadas (p < 0,001).
T17^(30)^	Contenido y clínica	Validado por embarazadas y especialistas. No se proporcionó más información. Uso asociado a mayor percepción de preparación para el parto.
T18^(31)^	Contenido y clínica	Alta satisfacción entre las primigrávidas (media de 9,92) Aumento significativo de los conocimientos sobre los signos clínicos tras la intervención

Subtítulo:IVC = Índice de Validación de Contenido; IC = Índice de Concordancia; CAP = Conocimiento Actitud Práctica.

## DISCUSIÓN

El análisis de las 18 TES mapeadas reveló un predominio de los recursos digitales, lo que refleja las tendencias contemporáneas en el campo de la educación en salud, con énfasis en una mayor accesibilidad, alcance de la información y posibilidad de actualización ágil de los contenidos^(23,24,31)^.

### Patrones Identificados (Patterns)

Se observó un patrón de lenguaje claro, accesible, sencillo y culturalmente adecuado, junto con el uso de ilustraciones instructivas para explicar o ejemplificar los signos de riesgo y los aspectos del parto. Algunas ETS utilizaron estrategias lúdicas como juegos y cómics^(15)^, narración de historias^(14,28,31)^, foros y canales de comunicación con profesionales de la salud, promoviendo el compromiso y fortaleciendo el vínculo entre las gestantes y los equipos de asistencia^(19,28,30)^.

### Avances Observados (Advances)

Se observaron avances en el rigor metodológico adoptado en la elaboración de las TES, con una sólida base en referencias teóricas actualizadas y la validación por parte de especialistas y/o mujeres embarazadas. Destacan las tecnologías desarrolladas a partir de las necesidades del público objetivo^(13,15,16,18,19,23)^, factor crucial para aumentar la aceptabilidad y la eficacia.

Además, la realización de validaciones clínicas reforzó la credibilidad de algunas tecnologías, apuntando resultados positivos, como el aumento del conocimiento sobre los signos de riesgo y el trabajo de parto^(14,31)^, una mayor percepción de la preparación para el parto^(30)^ y mejoras significativas en el CAP sobre la SHG^(12)^ y el parto^(22)^, lo que refuerza la credibilidad y favorece la replicabilidad de estas tecnologías.

### Brechas Identificadas (Gaps)

A pesar de los avances, se han identificado brechas importantes. Pocas tecnologías han abordado exclusivamente los signos de riesgo gestacional o del parto, siendo habitual la inclusión de estos contenidos de forma secundaria en propuestas educativas más amplias, lo que puede comprometer su eficacia en situaciones que requieren una toma de decisión rápida^(12,17,18,25,31)^.

También se ha identificado una escasez en el uso de recursos visuales realistas, como fotografías, potencialmente más eficaces en la representación de signos clínicos^(11)^. Además, la producción científica ha presentado desigualdades geográficas, concentrándose principalmente en las regiones Nordeste^(12, 13, 14,19, 20, 21,24, 25, 26,28,29,31)^ y Sudeste^(15,17,21,23,30)^, con poca representación de regiones como el Norte, caracterizada por mayores dificultades de acceso, diversidad cultural y peores indicadores obstétricos^(27)^.

Otro aspecto crítico fue la limitación en la representatividad de los participantes en los procesos de construcción y validación. La participación se concentró en las regiones donde se realizaron los estudios, lo que restringe la diversidad cultural y lingüística. Algunos autores informaron dificultades para reunir jueces de diferentes estados^(11)^ y una baja participación de los evaluadores invitados^(19,27)^. Además, la inclusión de adolescentes embarazadas (10 a 19 años) fue poco explorada, a pesar de su mayor vulnerabilidad a la desinformación y la inseguridad frente al embarazo y el parto^(13,14,22)^.

### Implicaciones Para la Práctica (Evidencia Para la Práctica)

Los hallazgos de esta revisión ofrecen información relevante para mejorar las políticas públicas dirigidas a la salud maternoinfantil, especialmente en el ámbito del Sistema Único de Salud (SUS). Las TES mapeadas presentaron evidencias prometedoras para mejorar la atención prenatal, promover el reconocimiento precoz de signos críticos y fortalecer la autonomía de las gestantes, de modo que su uso complementario a la actuación profesional puede contribuir a la reducción de los resultados maternos y neonatales adversos.

Así, la incorporación de estas tecnologías a las acciones educativas prenatales, a los protocolos de atención y a las estrategias de la Red Cigüeña y la Red Alyne constituye una oportunidad potencial para promover una atención más receptiva, inclusiva y basada en la evidencia.

La participación activa de la enfermería en el desarrollo y la aplicación de estas tecnologías refuerza el papel estratégico de la profesión en la promoción de la salud maternoinfantil y en la innovación educativa^(11, 12, 13, 14, 15, 16, 17, 18, 19, 20, 22, 23, 24, 25, 27, 28, 29, 30, 31)^. Las iniciativas que incorporan la accesibilidad, como la guía dirigida a mujeres con discapacidad visual^(16)^ y el vídeo con traducción al lenguaje de signos^(31)^, merecen ser destacadas como prácticas que promueven la equidad en el acceso a la información.

### Recomendaciones Para Futuras Investigaciones (Research Recommendations)

Se recomienda el desarrollo de nuevas tecnologías que amplíen el uso de recursos visuales realistas, como fotografías, asociadas a ilustraciones explicativas, respetando el contexto sociocultural y la sensibilidad del tema. Esta combinación puede potenciar la comprensión de los signos clínicos, sobre todo en públicos con diferentes niveles de alfabetización en salud^(9, 10, 11,22)^.

Para superar las limitaciones geográficas identificadas, es importante descentralizar los procesos de producción y validación de las tecnologías e involucrar a participantes de diferentes regiones del país. La inclusión de realidades diversas puede ampliar la aplicabilidad y la sensibilidad cultural de las herramientas educativas, contribuyendo a su eficacia en múltiples contextos. Para ello, se recomienda la formación de asociaciones entre instituciones de investigación de diferentes regiones, de carácter colaborativo o multicéntrico, favoreciendo la diversidad de la muestra y el intercambio metodológico. Las estrategias complementarias para captar participantes pueden incluir el uso de redes sociales, la difusión a través de asociaciones científicas y profesionales, la ampliación de los canales de contacto (correo electrónico, WhatsApp, llamadas telefónicas), el envío de recordatorios automáticos y la validación de la viabilidad de los plazos establecidos para las respuestas, con el fin de favorecer el compromiso, la corresponsabilidad y la retención de los participantes a lo largo del proceso.

En el caso de las adolescentes, se debe estimular la inclusión activa como participantes en los procesos de construcción y evaluación de las tecnologías^(22,28,29)^. Para ello, se sugiere la articulación con escuelas, unidades básicas de salud y servicios especializados en salud sexual y reproductiva, lo que puede facilitar el reclutamiento, el compromiso y la escucha cualificada de este grupo.

Por último, se refuerza la importancia de realizar validaciones clínicas rigurosas, con diseños sólidos y evaluación de resultados clínicos y conductuales, como el conocimiento, la actitud, la práctica y la adherencia a la atención prenatal. Estos resultados son fundamentales para respaldar la adopción de las tecnologías en la atención prenatal y su incorporación en políticas públicas basadas en la evidencia^(12,14,20,22,29)^.

### Limitaciones del Estudio

Aunque se utilizó la BDTD como fuente de literatura gris, la no inclusión sistemática de otros repositorios institucionales no indexados puede haber limitado la identificación de tecnologías educativas. Además, aunque las estrategias de búsqueda fueron amplias y se llevaron a cabo con rigor, es posible que la selección de bases y descriptores haya limitado la identificación de estudios relevantes.

## CONCLUSIÓN

Esta revisión de alcance ha mapeado 18 tecnologías educativas desarrolladas o utilizadas en el contexto de la atención prenatal brasileña para orientar a las mujeres embarazadas sobre los signos de riesgo gestacional y los signos indicativos del trabajo de parto.

Los resultados evidencian avances en la incorporación de recursos digitales, lenguaje accesible y validaciones rigurosas, con potencial para mejorar la atención prenatal y fortalecer la autonomía de las mujeres embarazadas. Sin embargo, persisten lagunas temáticas, metodológicas y geográficas, entre ellas el enfoque limitado y exclusivo de los signos críticos, la baja representatividad regional y la escasa inclusión de las adolescentes embarazadas.

Los hallazgos contribuyen a la ciencia de la enfermería al indicar vías para el desarrollo de tecnologías más específicas, inclusivas y clínicamente validadas, capaces de apoyar prácticas asistenciales basadas en la evidencia y de impactar positivamente en los resultados maternos y neonatales.

Se refuerza la necesidad de futuras investigaciones centradas en ampliar la diversidad cultural de los públicos involucrados y en el uso de recursos visuales más realistas para potenciar la eficacia de las intervenciones educativas.

## Data Availability

Todo el conjunto de datos que respalda los resultados de este estudio está disponible previa solicitud al autor correspondiente.
